# Angiotensin II receptor 1 antibodies associate with post-transplant focal segmental glomerulosclerosis and proteinuria

**DOI:** 10.1186/s12882-020-01910-w

**Published:** 2020-07-02

**Authors:** Mohammad Abuzeineh, Amtul Aala, Sami Alasfar, Nada Alachkar

**Affiliations:** 1 Department of Medicine, Division of Nephrology, The Johns Hopkins University School of Medicine, 600 N Wolfe St, Carnegie 344B, Baltimore, MD 21287 USA; 2 Beth Israel Deaconess Medical Center, Boston, MA USA

**Keywords:** Angiotensin II type 1 receptors (AT1R) antibody, Kidney transplant, Focal segmental Glomerulosclerosis, Proteinuria

## Abstract

**Background:**

Angiotensin II type 1 receptors (AT1Rs) are expressed on podocytes, endothelial and other cells, and play an essential role in the maintenance of podocyte function and vascular homeostasis. The presence of AT1R antibodies (AT1R-Abs) leads to activation of these receptors resulting in podocyte injury and endothelial cell dysfunction. We assessed the correlation between AT1R-Abs and the risk of post-transplant FSGS.

**Methods:**

This is a retrospective study, which included all kidney transplant recipients with positive AT1R-Abs (≥ 9 units/ml), who were transplanted and followed at our center between 2006 and 2016. We assessed the development of biopsy proven FSGS and proteinuria by urine protein to creatinine ratio of ≥1 g/g and reviewed short and long term outcomes.

**Results:**

We identified 100 patients with positive AT1R-Abs at the time of kidney transplant biopsy or proteinuria. 49% recipients (FSGS group) had biopsy-proven FSGS and/or proteinuria and 51% did not (non-FSGS group). Pre-transplant hypertension was present in 89% of the FSGS group compared to 72% in the non-FSGS group, *p* = 0.027. Of the FSGS group, 43% were on angiotensin converting enzyme inhibitors or angiotensin receptor blockers prior to transplantation, compared to 25.5% in the non-FSGS group, *p* = 0.06. Primary idiopathic FSGS was the cause of ESRD in 20% of the FSGS group, compared to 6% in the non-FSGS group, *p* = 0.03. The allograft loss was significantly higher in the FSGS group 63% compared to 39% in non-FSGS. Odds ratio and 95% confidence interval were 2.66 (1.18–5.99), *p* = 0.017.

**Conclusions:**

Our data suggest a potential association between AT1R-Abs and post-transplant FSGS leading to worse allograft outcome. Therefore, AT1R-Abs may be considered biomarkers for post-transplant FSGS.

## Background

Angiotensin II type 1 receptors (AT1Rs) are widely expressed across endothelial cells and podocytes. In previous reports, angiotensin II type 1 receptor antibodies (AT1R-Abs) have shown to be associated with vascular rejection of renal allografts in the absence of human leukocyte antigen (HLA) antibodies [1]. In animals, AT1R-Abs reported to be associated with malignant hypertension, preeclampsia and post-transplant focal segmental glomerulosclerosis (FSGS) [2]. In one case, a patient with positive AT1R-Abs presented with new onset collapsing FSGS and antibody-mediated rejection 1 month after renal transplantation [3]. Although the exact mechanism of injury in human is not known, it is thought that AT1R-Abs may cause activation of the AT1R receptors leading to podocyte injury, glomerular endotheliosis and proteinuria [4]. In animal models and cultured podocyte studies, the AT1R-Abs prevented the mRNA expression of the slit diaphragm molecules leading to proteinuria [5].

FSGS is a histopathologic diagnosis, classified as idiopathic (primary) or secondary. Post-transplant FSGS may be recurrent or de-novo in nature. Recurrent FSGS is very common with 30–40% recurrence rate post transplant [6]. Not all patients respond to treatment and some progress, leading to allograft loss [7].

The pathogenesis of recurrent FSGS is not well understood; however established data suggest that podocyte injury is secondary to circulating factor/s [8]. In a case report, recurrence of FSGS in renal allograft was reversed with complete resolution of proteinuria after re-transplantation into a different recipient [9]. Several factors have been investigated as potential causes of primary and recurrent FSGS [10], such as soluble urokinase type plasminogen activator (suPAR) [11] and cardiotrophin-like cytokine-1 (CLC-1) [12]. No one factor was validated in a large cohort. A recent study showed an association between pre-transplant AT1R-Abs in patients with primary FSGS and the risk of post-transplant recurrent FSGS [13].

In this study, we aim to assess the association between the presence of AT1R-Abs and the development of post-transplant FSGS and proteinuria.

## Methods

### Study population

The study was approved by the Institutional Review Board (IRB) at Johns Hopkins Hospital. This is a retrospective study that included all renal transplant recipients with AT1R-Abs concentrations ≥9 Units/ml, who were transplanted and followed at our center between 2006 and 2016. Data were collected throughout transplant period until last available follow up (ending December 2019) or until graft loss.

### AT1R-abs testing

AT1R-Ab testing was performed using quantitative ELISA (CellTrend GmbH, Luckenwalde, Germany) as described before [14], using sera collected at time of graft dysfunction. Briefly, serum was diluted of a 1:100, added to the 96-well polystyrene microliter plate coated with human AT1R derived from transfected Chinese hamster ovary cell extracts and incubated at 4 °C for 2 h. Following wash steps, a horseradish peroxidase-conjugated goat anti-human IgG detection antibody was added, followed by 1 h of incubation. 3,3′,5,5-tetramethylbenzidine (TMB) substrate was then added to the reaction mix [14]. Presence of antibody bound to AT1Rs was detected by a colorimetric change. A standard curve was generated to allow the quantitation of AT1R-Abs, using a control sample at varying concentrations (2.5, 5, 10, 20, and > 40 U/ml). If available, pre-transplant sera were also tested retrospectively. AT1R-Abs concentrations of ≥9 units/ml were reported as positive, in accordance with published data and established laboratory references [15].

### Outcomes definitions

The primary outcome was the development of FSGS lesion and/or proteinuria. FSGS was defined by renal allograft biopsy detection of FSGS lesions by light microscope (LM) or the presence of 20% or more effacement of the podocyte foot processes by electron microscope (EM) with or without FSGS lesions on light microscopy. In concordance with previous publications [16] and our clinical observation in particular in cases of early recurrent FSGS post kidney transplantation, the degree of podocyte effacement measured by EM of 20% or more is linked with significant proteinuria.

Other biopsy findings were classified using Banff classification system, utilizing the most updated Meetings’ Reports [17–20]. Our renal pathologists reviewed all the available biopsies.

Proteinuria was defined by urine protein creatinine (UPC) ratio of ≥1 g/g. We chose this degree of proteinuria in agreement with published literature, including our previous prospective studies that showed UPC ratio of ≥1 g/g is clinically relevant and warrants some type of intervention [6, 16].

Secondary outcomes were renal allograft loss, death-censored renal allograft loss, renal allograft survival time and all cause mortality. Renal allograft loss was defined as eGFR less than 15 ml/min/1.73 m^2^ for three or more consecutive months, re-transplantation, the need for long-term dialysis, or death. Death-censored allograft loss excluded patients who died with functional renal allograft.

### Therapeutic intervention

Rejection and post-transplant FSGS episodes were treated according to patients’ clinical presentation, pathological findings, degree of pathological chronicity and other individualized factors. Briefly, cell-mediated rejection episodes (CMR) were treated with either high dose of steroid or thymoglobulin. Antibody-mediated rejection (ABMR), and when indicated, was treated with plasmapheresis and intravenous immunoglobulin (IVIg) in addition to steroid, and rituximab in early cases. In most of rejection episodes, optimizing immunosuppressive medications was utilized.

Recurrent or de novo FSGS post-transplant episodes, when detected early before the development of significant sclerosis and IFTA, were treated with plasmapheresis followed by IVIg, in addition to ARB, or ACEi/or ARB in cases that are not candidate for plasmapheresis treatment. Also, some patients received rituximab and few received ACTHgel [6, 21].

### Statistical analysis

Baseline characteristics of the renal allograft transplant recipients were presented as proportions with percentage (%) or median with inter quartile range (IQR). Statistical analyses were performed using MedCalc 19.1.5.

Statistical differences were assessed by t-test for parametric data, Mann-Whitney test for non-parametric data and Pearson Chi square or Fisher’s exact test for categorical variables, as appropriate. In Kaplan Meier curve log-rank test was used to calculate the *p*-value. Forest plot was used to present secondary outcomes, with odds ratio and 95% confidence interval.

## Results

**Fig. 1:**
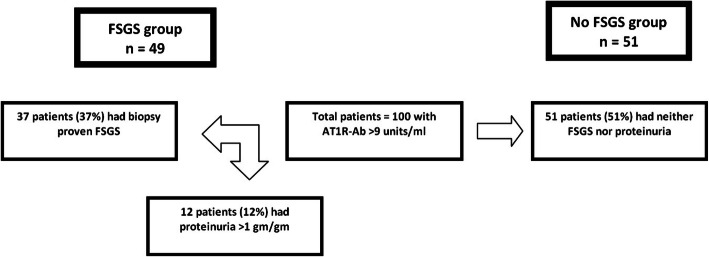
Primary outcome

We identified 100 kidney transplant patients with positive AT1R-Abs during the study period. Median follow up time was 64 (30–93) months. Out of 100 patients with AT1R-Abs, 37 patients (37%) were found to have biopsy proven FSGS and 12 patients (12%) had significant proteinuria of ≥1 g/g as measured by UPC ratio; total of 49 patients (49%) (FSGS group). Fifty-one patients (51%) did not have biopsy-proven FSGS or significant proteinuria (non-FSGS) group, Fig. 1.

Baseline characteristics are summarized in Table 1. The median age of the FSGS group was 51 years (44.75–61.25) and was 55 years (44.25–61) in non-FSGS group. Female patients were 57% in the FSGS group and 70% in the comparison group. Regarding race, 61% were white, 29% black, 10% other race in FSGS group compared to 70% white, 20% black, 10% other race in the comparison group. Median body mass index (BMI) was 25 (21–32) kg/m^2^ among FSGS group, and 27 (24–31.5) in the comparison group. Pre-transplant hypertension was present in 89% of the FSGS group, and 72% in the comparison group; the difference was statistically significant with *p* = 0.027. There was no statistical difference in the prevalence of pre-transplant diabetes mellitus between the two groups, 26.5% in FSGS group versus 23.5% in the comparison group. No patient in the cohort had hepatitis B or HIV, on the other hand chronic hepatitis C infection was present in 8% of FSGS group and in 4% in of the comparison group, *p* = 0.3719.

**Table 1:** Baseline characteristics

	FSGS *n* = 49	No FSGS *n* = 51	*p*-value
Median age, years (IQR^a^)	51 (44.75 to 61.25)	55 (44.25 to 61.00)	0.7667
Gender	Females 28/49 (57%)	Females 36/51 (70%)	0.1614
	Males 21/49 (43%)	Males 15/51 (30%)	
Race	White 30/49 (61%)	White 36/51 (70%)	
	Black 14/49 (29%)	Black 10/51 (20%)	0.5563
	Other 5/49 (10%)	Other 5/51 (10%)	
Median Body Mass Index (IQR^a^)	25 (21–32)	27 (24–31.5)	0.4593
Pre transplant hypertension	44/49 (89%)	37/51 (72%)	0.0279
Pre transplant diabetes	13/49 (26.5%)	12/51 (23.5%)	0.7289
Hepatitis B infection	0/49 (0%)	0/51 (0%)	NA
Hepatitis C infection	4/49 (8%)	2/51 (4%)	0.3719
HIV infection	0/49 (0%)	0/51 (0%)	NA
Use of ACEI^b^ or ARB^c^ prior to transplant	21/49 (43%)	13/51 (25.5%)	0.0668
Primary renal disease is primary FSGS	10/49 (20%)	3/51 (6%)	0.03
Primary renal disease is glomerular disease	10/49 (20%)	13/51 (25.5%)	0.5460

There were no statistically significant differences between FSGS group and the comparison group in the median age, gender, race, pre-transplant diabetes or use of ACEI or ARB prior to transplant. Pre transplant hypertension was more in the FSGS group than the comparison group, the difference was statistically significant. Primary FSGS as a cause of primary renal disease was higher in patients with FSGS to comparison group, the difference was statistically significant

^a^Inter Quartile Range

^b^Angiotensin converting enzyme inhibitors

^c^Angiotensin receptor blockers

Data are presented as proportions followed by percentage (%) unless otherwise mentioned

In the FSGS group, 43% were on angiotensin converting enzyme inhibitors (ACEi) or angiotensin receptor blockers (ARBs) prior to transplantation, compared to 25.5% in the comparison group, *p* = 0.06. The cause of ESRD was primary idiopathic FSGS in 20% of the FSGS group, compared to only 6% of the non-FSGS group; the difference was statistically significant with *p* = 0.03. Other primary glomerular diseases were the causes of ESRD in 20% of the FSGS group and 25.5% of the comparison group.

Allografts’ characteristics are summarized in Table 2. There was no statistical difference in the source of donated kidneys between the two groups; 69.5% were from living donors in the FSGS group, and 82.5% in comparison group (*p* = 0.12). Induction therapy was rabbit anti-thymocyte globulin (rATG) in 86% in FSGS group, and was 80% in the comparison group. 89% of patients in the FSGS group and 98% in the comparison group received standard maintenance immunosuppression (mycophenolate mofetil, tacrolimus and prednisone). 28.5% of patients in the FSGS group had one previous renal transplant, while 37% of the comparison group had previous one transplant, not statistically significant. Previous two or more transplants were noted in 20% in the FSGS group, and 10% in the comparison group. At the time of AT1R-Abs measurements, median serum creatinine and eGFR in FSGS group were 1.5 mg/dl (1.10–1.98) and 49 ml/min (36.5–63.5) and 1.2 mg/dl (1.00–1.90) and 49 ml/min (34–65) respectively in the non-FSGS group. Median UPC ratio in the FSGS group was 1.65 g/g (0.41–2.99) compared to 0.175 g/g (0.07–0.37) in the non-FSGS group, *p* < 0.00001. Donor-specific antibodies (DSAs), analyzed by flow-cytometry crossmatch test at the time of AT1R-Abs measurements, were negative in 28.5%, low level in 22.5% and positive in 49% in the FSGS group. In the comparison group, DSAs were negative in 37%, low-level in 23% and positive in 40%, *p* = 0.57. Biopsy- proven ABMR was present in 30.5% of the FSGS group compared to 25.5% of the comparison group, p = 0.57. Biopsy-proven CMR was present in 10% of FSGS group compared to 8% of the comparison group. Mixed cellular and antibody-mediated rejection was present in 2% of the FSGS group and in 4% of the comparison group. None of these differences in rejection rates were statistically significant. Median biopsy Banff scores for both FSGS and comparison groups who developed rejection episodes are summarized in supplementary Table 1.

**Table 2:** Allografts’ characteristics

	FSGS *n* = 49	No FSGS *n* = 51	*p*-value
Type of donation	DKT^a^: 15/49 (30.5%)	DKT: 9/51 (17.5%)	0.1291
	LKT^b^: 34/49 (69.5%)	LKT: 42/51 (82.5%)	
Induction immunosuppression	rATG^c^: 42/49 (86%)	rATG: 41/51 (80%)	0.4787
	Other: 7/49 (14%)	Other: 10/51 (20%)	
Maintenance immunosuppression	Standard^d^: 44/49 (90%)	Standard: 50/51 (98%)	0.0827
	Non standard: 5/49 (10%)	Non standard: 1/51 (2%)	
Previous one renal transplant	14/49 (28.5%)	19/51 (37%)	0.3559
Previous two or more renal transplants	10/49 (20%)	5/51 (10%)	0.1376
Median Cr at time of biopsy (IQR)	1.50 (1.10–1.98)	1.20 (1.00–1.90)	0.1770
Median eGFR at time of biopsy (IQR)	49 (36.5–63.5)	49 (34–65)	0.7113
Median proteinuria at time of biopsy (IQR)	1.65 (0.41–2.99)	0.175 (0.07–0.37)	< 0.00001
Presence of donor specific antibodies	Negative 14/49 (28.5%)	Negative 19/51 (37%)	0.5697
	Low positive 11/49 (22.5%)	Low positive 12/51 (23%)	
	Positive 24/49 (49%)	Positive 20/51 (40%)	
Biopsy proven ABMR^f^	15/49 (30.5%)	13/51 (25.5%)	0.5684
Biopsy proven CMR^g^	5/49 (10%)	4/51 (8%)	0.6800
Biopsy proven AMR and CMR (mixed)	1/49 (2%)	2/51 (4%)	0.5815

There were no statistically significant differences between FSGS group and comparison group in type of donation, induction immunosuppression, maintenance immunosuppression, number of previous transplants, presence of donor specific antibodies, presence of biopsy proven AMR or CMR or mixed rejection

^a^Deceased Kidney Transplant

^b^Living Kidney Transplant

^c^Rabbit anti-thymocyte globulin

^d^Standard immunosuppression: Mycophenolate mofetil, tacrolimus, prednisone. Non standard: any other

^e^HLA antibody testing was performed with pre and post transplant patients’ sera using the Luminex™ pooled HLA antigen (LMX), the phenotype bead assay (LMID) (Immucor-Lifecodes, Stamford, CT) and a single antigen panel (One Lambda, Canoga Park, CA)

^f^Antibody-mediated rejection

^g^Cell-mediated rejection

Data are presented as proportions followed by percentage (%) unless otherwise specified

**Fig. 2:**
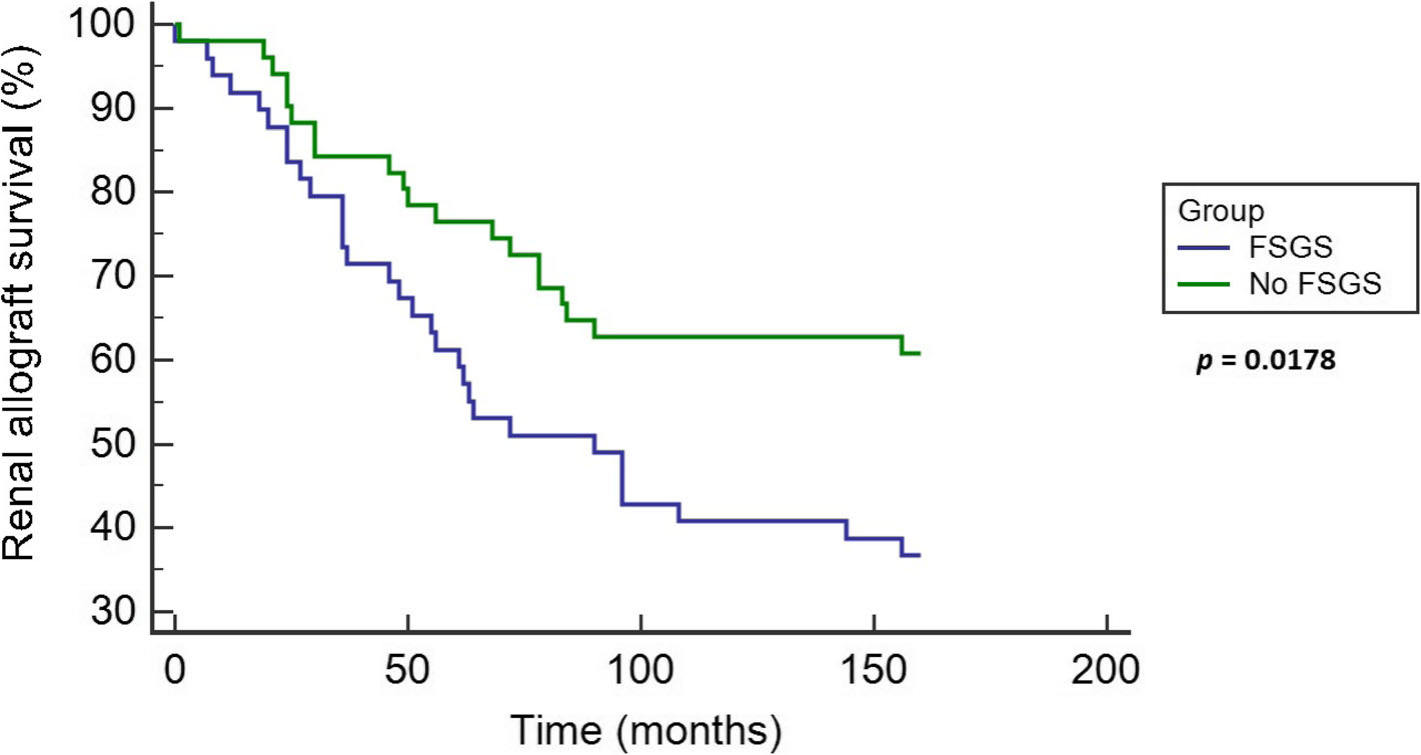
Kaplan-Meier curve for renal allograft survival. Renal allograft survival was 37% in FSGS group, 61% in the comparison group, *p* = 0.017. Mean allograft survival time (not shown) was comparable in FSGS group (54.3 months) and in comparison group (54.15), *p* = 0.99

**Fig. 3:**
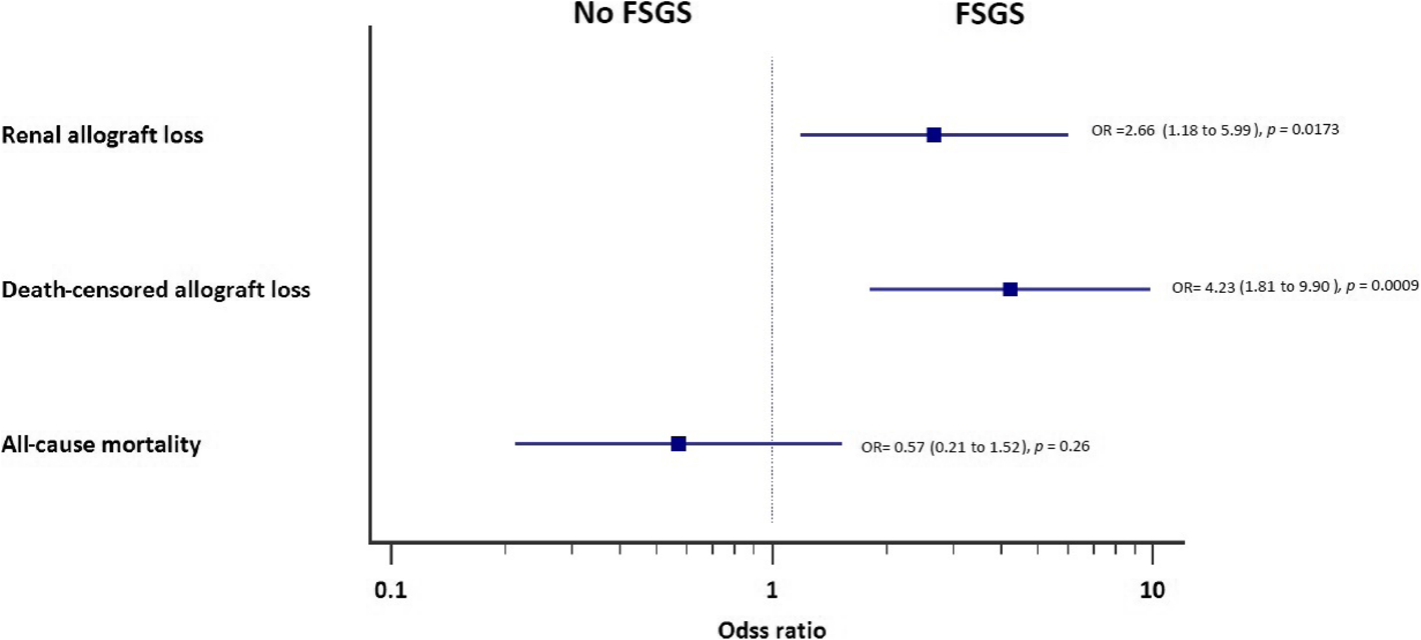
Forest plot for secondary outcomes. Renal allograft loss showing a statistically significant and higher odds ratio of 2.66 (1.18–5.99) in FSGS group than the non-FSGS group. Death-censored allograft loss showing even higher odds ratio of 4.23 (1.81–9.90) in FSGS group when compared to the other group. All-cause mortality was less in FSGS group than in the comparison group, however the difference is not statistically significant

Secondary outcomes are summarized in Table 3. Renal allograft loss was more prevalent in the FSGS group, 63% compared to 39% in the comparison group with odds ratio (95% confidence interval) of 2.66 (1.18–5.99) *p* = 0.017. Death-censored renal allograft loss was more observed in the FSGS group 59%, versus 25.5% in the comparison group, odds ratio (95% confidence interval) of 4.23 (1.81–9.90) *p* = 0.0009. All-cause mortality was less in the FSGS group compared to the other group (16.3 and 25.4% respectively), however the odds ratio and confidence interval 0.57 (0.21–1.52) was not statistically significant, *p* = 0.26. Figure 2 shows Kaplan-Meier curve comparing renal allograft survival of the patients in the two groups. Figure 3 shows Forest plot of secondary outcomes (renal allograft loss, death-censored renal allograft loss and all-cause mortality), presented as odds ration and 95% confidence interval, with calculated *p*-value.

**Table 3:** Secondary outcomes

	FSGS n = 49	No FSGS n = 51	OR (95% CI)	*p*-value
Renal allograft loss	31/49 (63%)	20/51 (39%)	2.6694 (1.1895 to 5.9905)	0.0173
Death-censored allograft loss	29/49 (59%)	13/51 (25.5%)	4.2385 (1.8130 to 9.9086)	0.0009
All-cause mortality	8/49 (16.3%)	13/51 (25.4%)	0.5704 (0.2130 to 1.5275)	0.2639
Mean graft survival time 95% Confidence Interval	52.48 months (38.94–66.02)	54.20 months (37.51–70.88)	Not applicable	0.8702

FSGS group showed higher renal allograft loss than the comparison group with statistically significant odds ratio as shown. This was more evident in the death-censored renal allograft loss with higher odds ratio and statistical significance. All-cause mortality was lower in FSGS group than the comparison group; however the difference was not statistically significant. Mean allograft survival was almost similar in both groups without statistically significant differences

Data are presented as percentage (%) unless otherwise mentioned

We assessed the correlation between the levels of AT1R-Abs and the risk of developing FSGS. There was no statistically significant difference in the levels of AT1R-Abs and the development of FSGS, Table 4.

**Table 4:** AT1R-Abs levels in both groups

AT1R-Abs Levels (units/ml)	FSGS *n* = 49	No FSGS *n* = 51	*p*-value
9–17	29/49 (59%)	25/51 (49%)	0.5779
17–40	13/49 (27%)	16/51 (31%)	
> 40	7/49 (14%)	10/51 (20%)	

We also evaluated mean and median time to event (development of biopsy-proven FSGS and/or proteinuria) among FSGS group who developed recurrent or de novo FSGS. Findings are summarized in supplementary Table 2.

## Discussion

Widely expressed on podocytes, angiotensin II type 1 receptors (AT1Rs) play an essential role in the maintenance of podocyte function, vascular homeostasis and several cellular and tissue functions in physiological state [13]. As demonstrated by animal models, AT1Rs hinder the mRNA expression of the slit diaphragm molecules, and their antagonists ameliorate proteinuria by preventing a reduction in the functional molecules of the slit diaphragm [5].

Despite the success of renal transplant in many FSGS patients, the risk of recurrence remains high and is estimated to be 30–40%. This risk can increase especially amongst patients with aggressive idiopathic (primary) FSGS or those with history of recurrence after previous transplant [6]. Recurrence often leads to allograft failure and loss. Several markers [8] were suggested to be associated with native kidney FSGS and post-transplant recurrence. Serum suPAR and apolipoprotein A1 (ApoA-1) are examples [22]; however additional factors are likely to exist. Given the wide expression of AT1Rs on podocytes, we evaluated the association of their antibodies (AT1R-Abs) and the risk of developing FSGS in renal transplant recipients. In our study, about half of the patients with positive AT1R-Abs were found to have biopsy-proven FSGS and/or significant proteinuria at the time of the AT1R-Abs detection, which could not be attributed to other causes. In those patients, renal allograft survival was significantly lower compared to those without FSGS or proteinuria (37% compared to 61%).

Mujtaba et al., tested pre-transplant sera of 28 patients with history of primary FSGS for anti-AT1R levels as a biomarker for risk of recurrence of FSGS [13]. Sera from 11 patients with polycystic kidney disease were used as controls. Twelve patients had biopsy-proven post-transplant FSGS recurrence at 1.5 months. AT1R-Abs levels in patients with FSGS were significantly higher in those who developed FSGS recurrence compared to those who did not. The authors concluded that pre-transplant AT1R-Abs levels might be a helpful biomarker in identifying patients at high risk of post-transplant FSGS recurrence [13]. In our study we assessed a larger sized cohort with positive AT1R-Abs and included patients with various causes of ESRD. Our study demonstrated that even without a primary diagnosis of FSGS, de novo FSGS can develop in the presence of AT1R-Abs. Our cohort included a total of 13 primary FSGS patients with positive AT1R-Abs, 10 of them developed recurrent FSGS.

We found that pre-transplant hypertension prevalence was higher amongst patients who developed post-transplant FSGS. Hypertension may be associated with secondary FSGS due to hyperfiltration. Despite the absence of statistical significance, pre-transplant use of ACEi or ARBs was more amongst AT1R-Abs positive patients who developed FSGS, which is possibly reflecting the higher prevalence of pre-transplant hypertension in that population.

In a retrospective study by Pascual et al., the authors compared the risk of recurrence of FSGS and other forms of glomerulonephritis, the rate of FSGS was lower in patients who received induction therapy with polyclonal rabbit anti-thymocyte globulin, compared with alemtuzumab and interleukin-2 receptor antagonist [23]. Another study showed mTOR inhibitors may cause post-transplant FSGS [24]. In our study, there were no statistically significant differences in the induction or and the maintenance immunosuppression between patients who developed FSGS and those who did not.

Secondary FSGS can also be a sequel of transplant rejection especially chronic, and can be associated with chronic transplant glomerulopathy and proteinuria. In this study, the biopsy- proven allograft rejection and presence/absence of DSAs were comparable in both FSGS and non-FSGS groups. However, it remains unclear why some patients with positive AT1R-Abs would develop post-transplant FSGS and some would not. This could be explained by other factors or possible “second hit” that is yet to be identified.

The real challenge resides in the management of patients with positive AT1R-Abs. Patients with acute non-HLA vascular rejection induced by AT1R-Abs are often treated with “all in” strategy, where pulse steroids, plasmapheresis, IVIG with or without rituximab are given. That could be related to delay in the diagnosis as not all patients are tested for non-HLA antibodies unless suspected in cases of negative DSAs. ARBs are added (if possible) which are likely of some benefit. However, in many cases, post-transplant FSGS may present with a sudden onset of nephrotic range proteinuria, a serial of plasmapheresis followed by IVIG in attempt to remove any other circulating factor/s or the AT1R-Abs may be indicated. In a case report, de novo collapsing FSGS in AT1R-Ab positive patient was successfully treated with plasmapheresis and losartan resulting in complete resolution of proteinuria [3]. However, the effectiveness of such measures is yet to be determined.

The strength of this study is that it is the first study to evaluate the association of AT1R-Abs and development of post-transplant FSGS in patients with various causes of ESRD. It provides an insight into possible pathogenesis of post-transplant FSGS whether recurrent or de novo. It also shows worse allograft outcomes when AT1R-Abs associate with FSGS. Our study has several limitations. The retrospective nature of this study may have potential bias. It involved more living kidney transplants and more female recipients, which do not represent the demographics of transplants in the U.S population. Additionally, there was a significant difference in pre-transplant hypertension between the two groups. The timing of the renal allograft biopsies and/or laboratory work up was not standardized and widely variable. Furthermore, the histopathology of renal biopsies, especially the degree of podocyte foot process effacement on EM can be variably estimated according to the reading pathologist. One more limitation is the fact that our study lacks a control group. Although, a control group will add a significant strength to our manuscript, unfortunately, only patients who we had a strong suspicion of having AT1R-Abs were tested. Therefore, we were unable to identify a matching group in whom the test was negative, and compared with our study cohort. However, although there is no matching control group, and in comparison to published data, AT1R-Abs seem to correlate highly with post-transplant FSGS, which many potentially considered permeability factors. Finally, we did not have data on APOL1 genetic variants amongst the black patients of the FSGS cohort, which could be a confounding factor. Nevertheless, our study is the first and largest to date with findings that may explain some cases of recurrent and de novo FSGS and proteinuria post-kidney transplant.

## Conclusion

AT1R-Abs may play a role in the pathogenesis and the development of recurrent and de novo FSGS and proteinuria post renal transplantation. The early detection may predict the risk for developing post-transplant FSGS; thus prompting closer follow up and initiating proper management. More studies are needed to confirm our findings of the role of AT1R-Abs in post-transplant FSGS and the impact of interventions targeting these antibodies on renal allograft outcome.

## Supplementary information

**Additional file 1: Table S1.** Median Banff scores for patients who developed rejection in FSGS and non-FSGS groups. **Table S2.** Mean and median time (months) to event among FSGS group with recurrent primary FSGS and de novo FSGS.

## Data Availability

All data generated or analyzed during this study are included in this published article.

## Abbreviations

AT1R: Angiotensin II type 1 receptor
AT1R-Abs: Angiotensin II type 1 receptor antibodies
FSGS: Focal segmental glomerulosclerosis
HLA: Human leukocyte antigen
suPAR: Soluble urokinase type plasminogen activator
ApoA-1: Apolipoprotein A1
eGFR: Estimated glomerular filtration rate
ESRD: End stage renal disease
LM: Light microscope
EM: Electron microscope
rATG: Rabbit anti-thymocyte globulin
DSA: Donor specific antibody
IQR: Inter Quartile Range
ABMR: Antibody-mediated rejection
CMR: Cell-mediated rejection
