# Supplementary material for: Angiotensin II receptor 1 antibodies associate with post-transplant focal segmental glomerulosclerosis and proteinuria
Source: BMC Nephrol. 2020 Jul 2;21:253. doi: 10.1186/s12882-020-01910-w (PMC7331243; doi:10.1186/s12882-020-01910-w)
Supplement: Supplementary file 1 — Additional file 1: Table S1. Median Banff scores for patients who developed rejection in FSGS and non-FSGS groups. Table S2. Mean and median time (months) to event among FSGS group with recurrent primary FSGS and de novo FSGS. [file 12882_2020_1910_MOESM1_ESM.docx]

Supplementary tables.

Supplementary table 1

| Banff score | FSGS group | Non FSGS group | p-value |
| --- | --- | --- | --- |
| g | 1.5 (1-2) | 1 (0.5-2) | 0.2584 |
| i | 0.5 (0-2) | (0.5-1.75) | 0.4715 |
| t | 1 (0-1) | 1 (0.5-1) | 0.9442 |
| v | 0 (0-0) | 0 (0-0) | 0.8965 |
| c4d | 0 (0-3) | 2.5 (0-3) | 0.2584 |
| ptc | 2 (0-3) | 3 (1-3) | 0.1738 |
| cg | 0 (0-3) | 0 (0-0) | 0.0307 |
| mm | 1 (0-2.5) | 0 (0-0) | 0.0071 |
| ci | 1.5 (0-2) | 0 (0-1) | 0.0051 |
| ct | 1.5 (0-2) | 0.5 (0-1) | 0.0059 |
| ti | 1.5 (0-2) | 1.5 (0-2) | 1 |
| cv | 0.5 (0-2) | 0 (0-1) | 0.2187 |
| ah | 0 (0-2) | 0 (0-0) | 0.3270 |
| Median Banff scores for patients who developed rejection in FSGS and non-FSGS groups. | | | |

Supplementary table 2

|  | Recurrent primary FSGS (n=10) | De novo FSGS  (n=39) | p-value |
| --- | --- | --- | --- |
| Mean time to event (SD) | 20 (22.5) | 37 (32.5) | 0.0663 |
| Median time to event (IQR) | 12 (3.75-30) | 25 (12-60.5) | 0.0574 |
| Mean and median time (months) to event among FSGS group with recurrent primary FSGS and de novo FSGS. | | | |
